# Dataset on usnic acid from *Cladonia substellata* Vainio (Lichen) schistosomiasis mansoni's vector control and environmental toxicity

**DOI:** 10.1016/j.dib.2017.12.068

**Published:** 2018-01-03

**Authors:** Hallysson Douglas Andrade de Araújo, Luanna Ribeiro dos Santos Silva, Williams Nascimento de Siqueira, Caíque Silveira Martins da Fonseca, Nicácio Henrique da Silva, Ana Maria Mendonça de Albuquerque Melo, Mônica Cristina Barroso Martins, Vera Lúcia de Menezes Lima

**Affiliations:** a Departamento de Bioquímica, Centro de Biociências, Universidade Federal de Pernambuco, Av. da Engenharia, S/N, Cidade Universitária, CEP 50670-420 Recife, PE, Brazil; b Departamento de Biofísica e Radiobiologia, Centro de Biociências, Universidade Federal de Pernambuco, Av. da Engenharia, S/N, Cidade Universitária, CEP 50670-420, Recife, PE, Brazil

## Abstract

This text presents complementary data corresponding to schistosomiasis mansoni's vector control and enviromental toxicity using usnic acid. These informations support our research article “Toxicity of Usnic Acid from *Cladonia substellata* (Lichen) to embryos and adults of *Biomphalaria glabrata*” by Araújo et al. [1], and focuses on the analysis of the detailed data regarding the different concentrations of Usnic Acid and their efficiency to *B. glabrata* mortality and non-viability, as also to environmental toxicity, evaluated by *A. salina* mortality.

**Specifications Table**

**Table:** 

Subject area	*Chemistry, Biology*
More specific subject area	*Natural products biochemistry*
Type of data	*Table and figures*
How data was acquired	*Stereoscopic microscope (Wild M3B, Heerbrugg, Switzerland)*
Data format	*Analyzed*
Experimental factors	*Usnic acid purification fromCladonia substellatalichen*
Experimental features	*Adult and embryonicB. glabrataunviability and mortality tests andA. salinamortality assay over Usnic Acid treatments were evaluated*.
Data source location	*Recife, Brazil.*
Data accessibility	*Data found in this article*

**Value of the data**

• The data provide supporting evidence from Araújo et al. [1] regarding the different effectiveness of usnic acid over embryonic stages non-viablity and adult mollusks mortality of *B. glabrata*.
• Data shows the sensitivity profile of the *B. glabrata* embryos to the usnic acid along the different concentrations used.
• Data from different times allow us to infer a possible time range to obtain effective results in the population control of *B. glabrata* adult mollusks.
• Usnic acid was less toxic to *A. salina* than Niclosamide at concentrations of effective molluscicidal activity.

## 1 Data

Table 1 shows *B. glabrata* embryos non-viability after different treatments with Usnic acid.

**Table 1:** *Biomphalaria glabrata* embryos unviability under different treatments with usnic acid.

**Treatments**		**Unviable according to Embryonic Stage (%)**			
		**Blastula**	**Gastrula**	**Trocophore**	**Veliger**
**Control 1**		1.3±0.57	0.3±0.6	1.0±1.0	1.0±1.0
**Control 2**		2.7±0.57	0.7±1.2	1.7±1.5	2.3±2.3
**1 µg mL^-1^Niclosamide**		100.0±0.0	100.0±0.0	100.0±0.0	100.0±0.0
**Usnic acid ( µg mL**^**-1**^**)**	1	1.7±1.15	2.7±3.8	4.7±2.5	10.0±4.0
	1.5	51.0±6.0	11.0±3.5	6.3±2.5	14.7±9.3
	2	100.0±0.0	22.7±9.2	8.0±4.4	25.0±7.9
	2.5	–	32.0±7.0	7.3±2.5	34.3±2.9
	3	–	36.3±16.1	14.0±6.6	49.3±17.2
	3.5	–	50.0±8.2	15.7±5.5	72.0±3.6
	4	–	72.7±3.5	16.7±1.2	89.3±6.6
	4.5	–	100.0±0.0	24.7±2.3	93.7±5.7
	5	–	–	29.0±1.0	93.7±4.9
	5.5	–	–	94.3±3.8	97.0±5.2
	6	–	–	100.0±0.0	100.0±0.0

Values expressed as mean (± standard deviation) of eggs mass. Control 1: water only; Control 2: 0.5% DMSO.

**Fig. 1:**
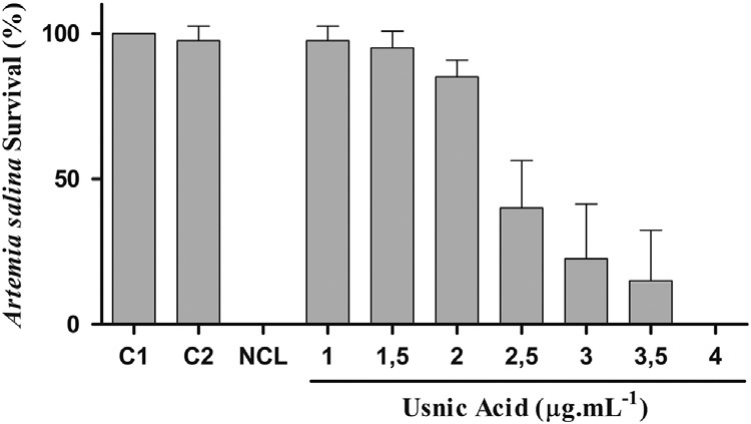
Usnic acid mortality on adult snails of *B. glabrata*. Control 1 (C1): water only. Control 2 (C2): 0.5% DMSO. NCL: niclosamide 1 µg mL^-1^. Usnic acid concentrations in µg mL^-1^.

Fig. 1 shows adult *B. glabrata* mortality after 24 hours and 7 days Usnic Acid treatment in comparison with niclosamide.

**Fig. 2:**
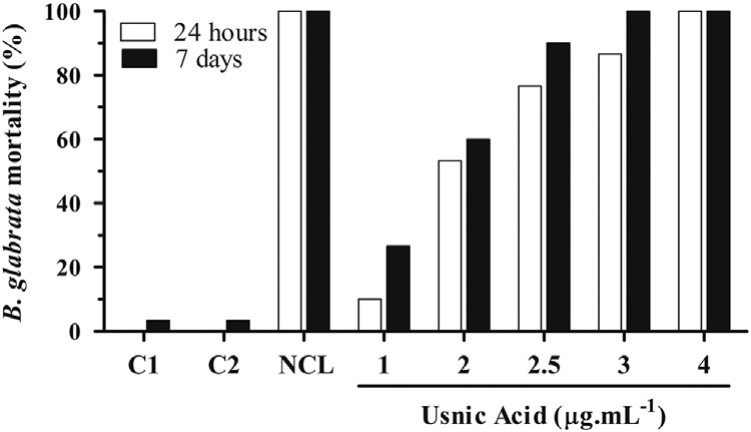
*Artemia salina* 7-day survival after 24-h different treatments. C1 (Control 1): Water only. C2 (Control 2): 0.5% DMSO. (NCL): 1 µg mL^-1^ niclosamide. Usnic acid concentrations in µg mL^-1^.

Fig. 2 demonstrates environmental toxicity caused by the usnic acid in comparison with the commercial drug, niclosamide, at concentrations of effective molluscicidal activity.

## 2 Experimental design, materials and methods

### 2.1 *B. glabrata* embriotoxicity assay

The embryotoxicity assay was performed according to the methodology described by Rapado et al. [2]. Briefly, colorless polyethylene pieces (10×10 cm) were placed on the water surface of the aquarium to collect egg masses. Those one deposited were separated with the help of a stereoscopic microscope (Wild M3B, Heerbrugg, Switzerland) and analyzed according to Kawano et al. [3] regarding to their viability.

Embryo stage was identified after cleavage of the eggs as follows: first (blastula: 0–15 h), second (gastrula:24-39 h), third (trocophore: 48–87 h) and fourth cleavage (veliger: 96–111 h). Groups of 100 embryos (n=100) from each stage were selected and deposited in petri dishes with 10 mL of Usnic Acid solutions (0.5% DMSO) at different concentrations, as follows: blastula (1, 1.5, and 2 µg mL^-1^); gastrula (1, 1.5, 2, 2.5, 3, 3.5, 4, and 4.5 µg mL^-1^) trocophore and veliger (1, 1.5, 2, 2.5, 3, 3.5, 4, 4.5, 5, 5.5, and 6 µg mL^-1^). Two negative control groups were used: water only (Control 1) and 0.5% DMSO (Control 2). A positive control group with 1.0 µg mL^-1^ niclosamide (NCL) was used.

All groups were exposed for 24 h, then embryos were washed and placed in clean plates with filtered and dechlorinated water and observed using microscope during 7 consecutive days in order to check their positive (hatch) or negative (death or malformation) viability, as described in Table 1. Two independent experiments were performed in triplicate.

### 2.2 Adult *B. glabrata* toxicity assay

*B. glabrata* toxicity assay was performed according to the WHO [4] recommended method. Pigmented *B. glabrata* (10 and 14 mm) were placed in individual containers (180 mL water) and observed for seven consecutive days to check sexual maturity. Eight groups (n=10) were used, which were designed similarly to the embriotoxicity experiment: Negative Controls - Filtered and dechlorinated water only (control 1), 0.5% DMSO Water (control 2); Positive Control (1.0 µg mL^-1^ NCL) and usnic acid (1, 2, 2.5, 3 and 4 µg mL^-1^) during 24 h.

After exposure, the living mollusks were transferred to vessels containing 1000 mL of filtered and dechlorinated water, fed and monitored daily for 7 days, as described in Fig. 1. Cephalopodal mass retraction into the shell, loss of hemolymph, discoloration of shell and absence of beats in the pericardial cavity were used as mortality criteria. Two independent experiments were performed in triplicate.

### 2.3 Environmental toxicity test using *A. salina*

*A. salina* encysted eggs were placed in a beaker with 500 ml seawater (pH 8.0) and constant aeration at room temperature (25±3 °C) for 48 h. After hatching, the larvae were collected and splitted in experimental groups (*n*=10) with the help of a stereomicroscope (Wild M3B, Heerbrugg, Switzerland) as follows: Negative Controls (C1: sea water only; and C2: 5% DMSO in sea water) and Usnic Acid (at 1, 1.5, 2, 2.5, 3, 3.5, and 4 µg mL^-1^) for 24 h at 25±3 °C according to the procedure described by Meyer et al. [5] (Fig. 2). Two experiments were performed in quadruplicate and assessments of mortality and survival of larvae were carried out by observation of mobility with the help of a stereomicroscope.
