# Supplementary material for: Dataset on usnic acid from Cladonia substellata Vainio (Lichen) schistosomiasis mansoni's vector control and environmental toxicity
Source: Data Brief. 2018 Jan 3;17:288–91. doi: 10.1016/j.dib.2017.12.068 (PMC5988437; doi:10.1016/j.dib.2017.12.068)
Supplement: Supplementary file 1 — Supplementary material [file mmc1.pdf]

Conflicts of Interest Statement

Manuscript title: Dataset on usnic acid from *Cladonia substellata* Vainio (Lichen) schistosomiasis mansoni's vector control and environmental toxicity.

The authors whose names are listed immediately below certify that they have NO affiliations with or involvement in any organization or entity with any financial interest (such as honoraria; educational grants; participation in speakers' bureaus; membership, employment, consultancies, stock ownership, or other equity interest; and expert testimony or patent-licensing arrangements), or non-financial interest (such as personal or professional relationships, affiliations, knowledge or beliefs) in the subject matter or materials discussed in this manuscript.

Author names: **Hallysson Douglas Andrade de Araújo**

Luanna Ribeiro dos Santos Silva

Williams Nascimento de Siqueira

Caíque Silveira Martins da Fonseca

Nicácio Henrique da Silva

Ana Maria Mendonça de Albuquerque Melo

Mônica Cristina Barroso Martins

Vera Lúcia de Menezes Lima

The authors whose names are listed immediately below report the following details of affiliation or involvement in an organization or entity with a financial or non-financial interest in the subject matter or materials discussed in this manuscript. Please specify the nature of the conflict on a separate sheet of paper if the space below is inadequate.

Author names:

This statement is signed by all the authors to indicate agreement that the above information is true and correct (*a photocopy of this form may be used if there are more than 10 authors*):

Author's name (typed)	Author's signature	Date
Hallysson Douglas A. de Araújo	Hallysson Douglas Andrade de Araújo	11/01/2018
Luanna Ribeiro dos Santos Silva	Luanna Ribeiro Santos Silva	15/01/2018
Williams Nascimento de Siqueira	Williams Nascimento de Siqueira	12/01/2018
Caíque Silveira Martins da Fonseca	Caíque Silveira Martins da Fonseca	18/01/2018
Nicácio Henrique da Silva	Nicácio Henrique da Silva	12/01/2018
Ana Maria Mendonça de A. Melo	Ana Maria Mendonça de A. Melo	12/01/2018
Mônica Cristina Barroso Martins	[illegible]	15/01/2018
Vera Lúcia de Menezes Lima	Vera Lucia M. Lima	15/01/2018
